# Out-of-Hospital Cardiac Arrest Ambulance Delay Zones and AED Placement in a Southern Brazilian City

**DOI:** 10.3390/ijerph22020173

**Published:** 2025-01-27

**Authors:** Marcos Rogério Bitencourt, Mariá Romanio Bitencourt, Lincoln Luís Silva, Amanda Gubert Alves dos Santos, Pedro Iora, José Anderson Labbado, Mauricio Medeiros Lemos, Luiz Gustavo de Paulo, Júlia Loverde Gabella, Juliana Lourenço Lopes Costa, Hideky Ikeda Dolci, Vinicius Giacomin, Sandra Marisa Pelloso, Maria Dalva de Barros Carvalho, Luciano de Andrade

**Affiliations:** 1 Post Graduate Program in Health Sciences, State University of Maringá, Maringá 87020-900, Paraná, Brazil; lincoln.luis@grupointegrado.br (L.L.S.); andersonlabbado@hotmail.com (J.A.L.); maulemostorax@gmail.com (M.M.L.); vgiacomin9@gmail.com (V.G.); smpelloso@uem.br (S.M.P.); mdbcarvalho@gmail.com (M.D.d.B.C.); landrade@uem.br (L.d.A.); 2 Department of Medicine, State University of Maringá, Maringá 87020-900, Paraná, Brazil; romanio.maria@gmail.com (M.R.B.); pedroiora85@gmail.com (P.I.); julialgabella@gmail.com (J.L.G.); 3 Department of Emergency Medicine, Duke University School of Medicine, Durham, NC 27708, USA; 4 Department of Health Sciences, Centro Universitário Integrado, Campo Mourão 87309-701, Paraná, Brazil; amanda.gubert@grupointegrado.br; 5 Department of Medicine, Centro Universitário de Maringá, Maringá 87050-900, Paraná, Brazil; lgdpaulo@gmail.com (L.G.d.P.); julianalourenco2501@gmail.com (J.L.L.C.); hidekyikedadolci@gmail.com (H.I.D.)

**Keywords:** out-of-hospital cardiac arrest, cardiopulmonary resuscitation, automated external defibrillators, spatial analysis, computer simulation

## Abstract

Out-of-hospital cardiac arrests (OHCAs) have high mortality rates, worsened by limited access to automated external defibrillators (AEDs). This study analyzed OHCA response times, identified areas with prolonged ambulance travel times, and proposed optimal AED locations in a medium-sized city in southern Brazil. Data from 278 non-traumatic OHCA cases (2019–2022) in patients over 18 years old, with ambulance response times under 20 min, were included. Spatial survival analysis assessed the probability of exceeding the recommended 5-min (300 s) ambulance response time. The maximal covering location problem identified 100 strategic AED sites within a 150-s reach for bystanders. AED and ambulance travel times were compared using the Wilcoxon test (*p* < 0.01). Defibrillation occurred in 89 cases (31.01%), and bystander CPR was performed in 149 cases (51.92%). Despite these efforts, 77% of patients died. The median ambulance response time was 11.63 min, exceeding 5 min in most cases, particularly at peak times like 11 a.m. AED placement in selected locations could cover 76% of OHCA occurrences, with a mean AED travel time of 320 s compared to 709 s for ambulances. Strategic AED placement could enhance early defibrillation and improve survival outcomes.

## 1. Introduction

Out-of-hospital cardiorespiratory arrest (OHCA) is a frequent medical emergency with a survival rate of less than 10% [1,2]. Survival rates at hospital discharge after OHCA are approximately 3% in Asia, 6.8% in North America, 7.6% in Europe, and 9.7% in Australia, posing an immense challenge for health systems worldwide [3,4].

Data from the United States show that approximately 350,000 cases of OHCA occur every year [1]. In Brazil, it is estimated that 200,000 cases of cardiac arrest occur annually, half of which take place in an out-of-hospital setting [5].

In 3917 of the 5570 Brazilian municipalities, the Mobile Emergency Care Service (SAMU) is responsible for the emergency management of OHCA [6]. In the case of an emergency, individuals can contact SAMU by making a toll-free call to 192 and will be asked to provide information about the patient’s condition and geographical location. Based on this information, a physician conducts a severity assessment, activates the care team, and instructs the caller on how to perform cardiopulmonary resuscitation (CPR) until the ambulance arrives to provide specialized care [7].

The first phase of cardiac arrest, known as the electrical phase, lasts approximately 4 min and is characterized by a shockable rhythm. In this phase, early defibrillation can increase the chances of survival [8]. The optimal window for utilizing an AED is within 3–5 min of the collapse [9,10].

Although the survival rate of OHCA is typically less than 10%, it can reach values as high as 70% in settings where AEDs are rapidly available, associated with bystander CPR, within 5 min of the collapse [1,11,12,13]. Spatial analysis can be a valuable tool in the strategic allocation of AEDs, improving the response to OHCA before ambulance arrival [14,15,16,17,18].

Understanding the particularities of OHCA cases is fundamental to effectively allocate health resources and improve survival rates. Optimizing the assistance provided by bystanders requires the placement of easily accessible, public-access AEDs close to OHCA sites [19,20]. Several international studies underscored the importance of CPR [21,22] and early defibrillation performed by bystanders to OHCA outcomes [8,10,23,24]. To the best of our knowledge, no studies have been conducted in Brazil to guide the allocation of public-access AEDs to facilitate bystander use and early defibrillation. Considering the above, this study aimed to analyze the SAMU OHCA response time, seeking to identify areas with longer response times and define the optimal location of AEDs to reduce the defibrillation time in a medium-sized city in southern Brazil.

## 2. Materials and Methods

### 2.1. Study Design

This is a cross-sectional, observational study using geospatial analysis to analyze the response time of SAMU ambulances to OHCA sites in Maringá, Paraná, Brazil, from 2019 to 2022. The procedures followed Strengthening the Reporting of Observational Studies in Epidemiology (STROBE) guidelines [25].

### 2.2. Study Site

Maringá is one of the 399 municipalities of Paraná State, Southern Brazil. It has a population of 409,657 inhabitants and a geographical area of 487,012 km^2^. The Municipal Human Development Index (MHDI) is 0.808, ranking 23rd among the 5570 Brazilian municipalities [26]. Furthermore, it is in 34th position among municipalities with the largest vehicle fleet in Brazil, with 312,553 vehicles, which represents a density of 1.31 inhabitants per vehicle.

**Figure 1:**
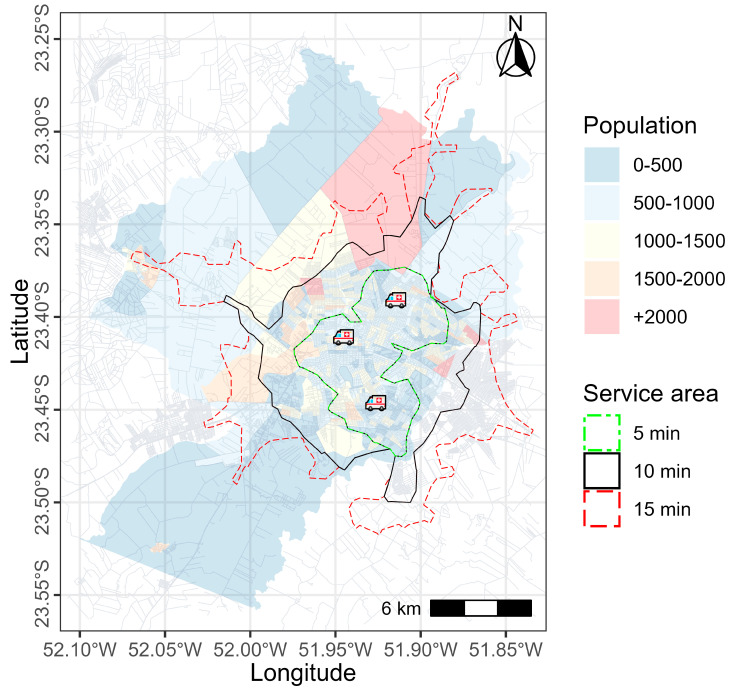
Population density, location, and service area coverage of SAMU units.

Figure 1 displays the population distribution of Maringá in each census sector, the location of the SAMU bases (represented by the figure of the ambulance), and the ambulance coverage in 5 (300 s), 10 (600 s), and 15 (900 s) min.

### 2.3. Data Collection

SAMU service records of OHCA cases occurring between 2019 and 2022 in Maringá were retrieved and exported to a Microsoft Excel^®^ spreadsheet. The following variables were extracted: caller ID, address, sex, age, date and time, time at which the call was transferred from the emergency call technician to the medical control physician, time at which the physician activated the emergency team, time at which the fleet dispatcher activated the emergency team, time of ambulance arrival at the scene, time of ambulance departure from the location, time of ambulance arrival at the destination, time of death, whether bystanders performed CPR, presence of shockable rhythm, time duration CPR by the SAMU team.

In this study, response time was defined as the interval between the receipt of the emergency call by the SAMU dispatch center and the arrival of the ambulance at the OHCA scene. While this period includes dispatch time (the interval between receiving the call and dispatching the ambulance) and travel time (the interval between dispatch and arrival on scene), we did not analyze these components separately. Given the criticality of out-of-hospital cardiac arrest (OHCA), where defibrillation is most effective in the first few minutes, we focused on total response time. Specifically, we sought to minimize all components of response time to ensure arrival on scene within 5 min, aligning with guidelines for early defibrillation and effective resuscitation.

### 2.4. Inclusion and Exclusion Criteria

The study included all records of non-traumatic OHCA among individuals aged 18 or older that occurred between 2019 and 2022. The analysis was limited to cases where emergency medical services arrived at the scene within 20 min of the OHCA event [27]. This time restriction was based on the well-established scientific principle that the efficacy of defibrillation, a fundamental intervention for reversing cardiac arrest, declines significantly after 10–15 min [28]. Cases with response times greater than 20 min were excluded due to geographical limitations in ambulance coverage and the potential for data entry errors in prolonged response situations. By focusing on cases with response times under 20 min, the study enables a more precise evaluation of the optimal therapeutic window for defibrillation, thereby ensuring the clinical significance of the findings.

### 2.5. Data Analysis

The maximum time to defibrillation was defined as 5 min. The analysis was divided into five steps. Thus, it was possible to evaluate the SAMU response time and identify the most suitable public places for the installation of AEDs. In the first step, descriptive statistics were applied to characterize the study population. Categorical variables are presented as absolute and relative frequencies, and continuous variables as median, minimum, and maximum values. The second step consisted of an isochronous analysis of the ambulance coverage area under ideal conditions, without complications, for response times of 5, 10, and 15 min from SAMU bases [29]. Isochronous analysis allows mapping accessibility areas and identifying what distances can be traveled in a given time using a given means of transport [30].

In the third step, a spatial survival analysis with Bayesian statistics was performed to determine the probability of an ambulance taking longer than the recommended threshold to arrive at the OHCA site. In the fourth step, a maximal covering location problem (MCLP) was applied to identify the most suitable public places for AED installation. In the fifth and final step, boxplots and the Wilcoxon test for paired samples were used to compare ambulance response times with and without AED implementation.

### 2.6. Spatial Survival Analysis

Spatial survival analysis can be used to model the occurrence of survival events over time, accounting for the influence of geographical location on observations [31]. When survival data are spatially referenced, spatial variation in survival may be of scientific interest [31]. The analysis can also determine correlations between different geographical areas and the probability of exceeding an established response time.

OHCA sites were georeferenced into latitude and longitude coordinates. The response time was calculated in seconds, encompassing the beginning of the call to the arrival of the ambulance at the scene. The response time variable was treated as censored when greater than 5 min. This cut-off was defined according to AHA recommendations [10].

Given that survival analysis requires defining the survival time, in this study, this variable was replaced with the response time. Thus, longer survival times indicate a delay in ambulance arrival. The probability of the ambulance exceeding the cut-off time was calculated from the posterior exceedance probability (PEP) [31]. The PEP represents the likelihood of an event occurring above a reference value, which, in this analysis, was set at 5 min. A probability between 0.8 and 1 indicates that, in more than 80% of occurrences, the ambulance does not arrive in less than 5 min. The model requires the inclusion of covariates such as call time and spatial location to estimate the probability. A Monte Carlo Markov chain (MCMC) algorithm was used to simulate data from the database, affording a robust result based on posterior elements [31]. To determine the posterior elements, we used a model for the prior where the dependent variable belonged to the surv class, and the independent variables were the intensities of SAMU call periods over 24 h. Additionally, beta and omega were fixed at a mean of 0 and a standard deviation of 100 and 10, respectively. Finally, eta was determined as values from a Gaussian distribution with a logarithmic mean of values between 1 and 1000 and a standard deviation of 0.5. These values were chosen after visually verifying the stability of the posterior results. Subsequently, the MCMC call was performed using the BsplineHaz function with a grid size of 500 m, and the control parameters nits (number of iterations), burn (initial discarded iterations), and thin (alternating iterations between retained and discarded) were set to 50,000, 10,000, and 490, respectively. With the results of the MCMC, it was possible to determine the relative risk of the ambulance exceeding an arrival time of 5 min by harmonic regression, considering the time of day with the greatest number of calls in a 24-h period [32,33]. The analysis was performed using the *Spatsurv* package of R software version 4.0 [34].

### 2.7. MCLP

For analysis of the coverage potential of AEDs, the MCLP model was applied using the Gurobi Optimizer https://www.gurobi.com/ (accessed on 15 July 2024), available in the Python language. MCLP is a mathematical optimization problem that determines the best location of facilities among a set of candidates. It aims to maximize the coverage of demand points while taking into account resource constraints. In the analysis, the number of available AEDs was gradually increased until the limit of potential coverage was reached [33,34]. Coverage encompassed distances to and from the AED location that could be reached by bystanders in 2.5 min, resulting in a time to defibrillation of up to 5 min [10].

For the analysis, a grid composed of 500 m^2^ cells was generated. The number of events occurring in each cell was counted, and it was assumed that events occurred at the centroid of cells. Thus, accessibility to AED was evaluated based on the distance from the centroid to the potential AED location. Additionally, the response time of events was averaged per cell. This procedure precludes individual calculation for each occurrence, focusing on centroid coverage. The model included 624 potential public establishments and considered a pedestrian speed of about 10 km/h [35], with a cut-off of 2.5 min from the centroid. A detailed description of the approach was deposited in an online repository.

### 2.8. Violin Plot Analysis

After MCLP analysis, the travel times of bystanders to the AED were determined for comparison with SAMU response times using the violin plot. The normality of the data was tested, and it was found that neither SAMU response times nor bystander displacement times followed a normal distribution. Therefore, median values of the evaluated times were compared using the Wilcoxon test. Values were considered statistically significant at *p* < 0.05.

### 2.9. Preparation of Thematic Maps

**Figure 2:**
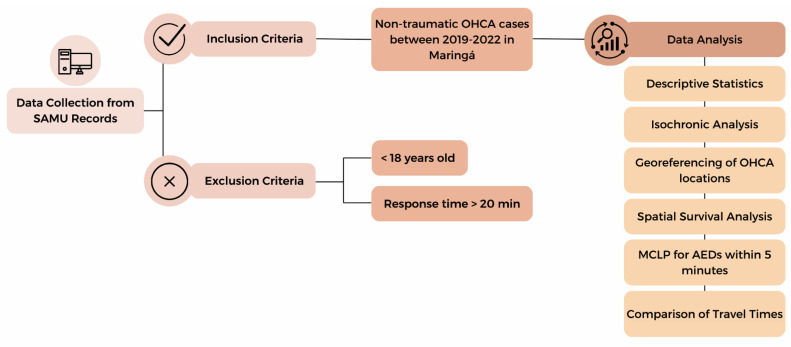
Methodological workflow of this study.

Descriptive analysis and spatial survival analysis with Bayesian statistics were performed in R Studio software (version 2023.12.0-369) using dplyr and spatsurv packages. Isochronous and MCLP analyses were conducted in Python using Gurobi software (version 11.0). Figure 2 summarizes the methodology used in this study.

## 3. Results

From 2019 to 2022, there were 476 calls for OHCA. Complete information was available for 287 (60.29%) calls, which were thus analyzed in this study. Most of the calls (168, 58.54%) were requested for male patients, and the median age of individuals was 69 years. In 89 (31.01%) OHCA occurrences, a shockable rhythm was identified, and defibrillation was performed. The median response time was 11.63 min, and the median CPR time was 30 min. Bystanders initiated CPR in 149 (51.92%) occurrences. In 179 (62.37%) occurrences, SAMU took more than 10 min to arrive, and, in 221 (77%) occurrences, the outcome was death. Shockable rhythm was present in 50 (56.18%) of the OHCA with EMS arrival time over 10 min (Table 1).

**Figure 3:**
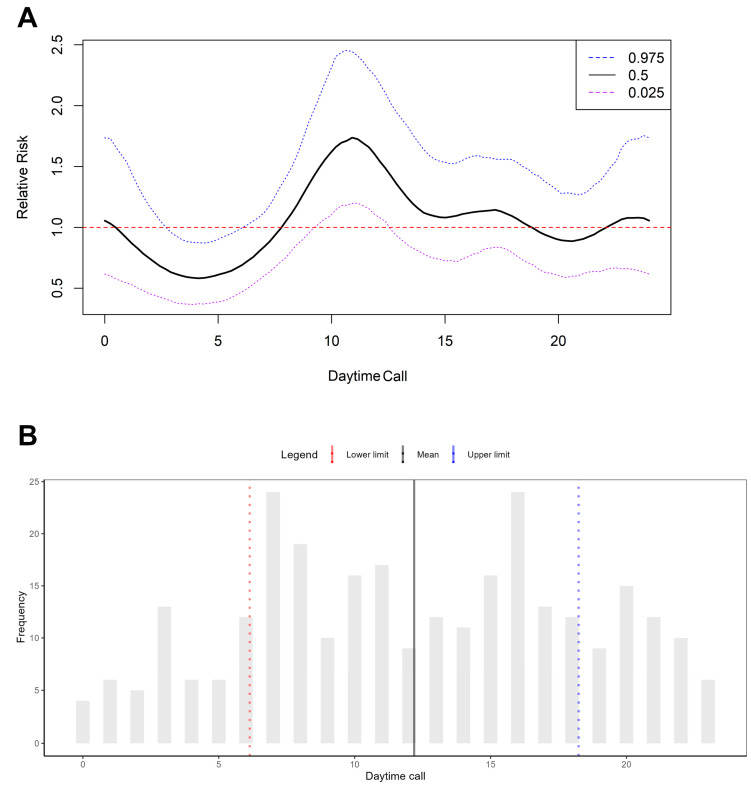
Relative risk and frequency of daily out-of-hospital cardiac arrest (OHCA) occurrences. (**A**) Relative risk of occurrences attended by SAMU over a 24-h period. (**B**) Frequency of daily occurrences across the 24-h period.

Figure 3A shows the relative risk for occurrences attended by SAMU over a 24-h period. The lowest-risk period was near 4:00 h, with a risk of 0.58. This finding indicates a 42% reduction in the risk of the response time exceeding 5 min, as represented by the median line and confidence interval below the threshold of 1.0. By contrast, at about 11:00 h, the risk achieved its maximum value (1.73). In other words, there was a 73% increase in the risk of the response time exceeding 5 min. Figure 3B shows the frequencies of daily occurrences, with peaks at 6:00 and 16:00 h and mean values near 12:00 h. The hour with the lowest occurrence was 00:00 h.

**Figure 4:**
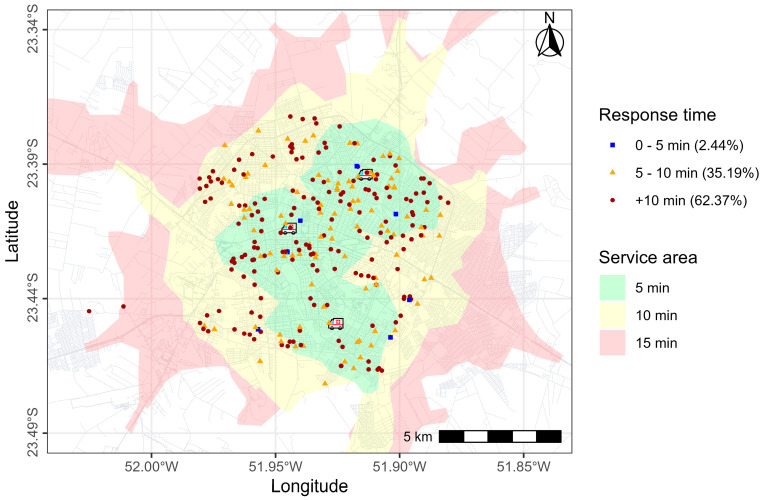
Spatial distribution of out-of-hospital cardiac arrest (OHCA) occurrences attended by SAMU and ambulance service area.

**Figure 5:**
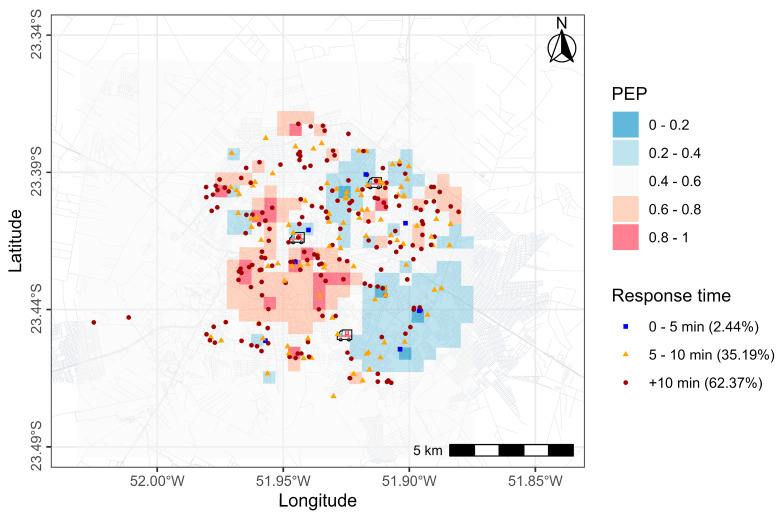
Posterior exceedance probability (PEP) areas of SAMU.

Figure 4 shows the distribution of occurrences attended by SAMU and isochrone and spatial survival maps. In Figure 3, dots represent the occurrence sites, and colors represent the response times. Polygons of different colors represent the service area, that is, areas with SAMU travel times of 5, 10, and 15 min. The majority of occurrences were located in the municipality’s central region. Although the 5 min coverage area was large, in practice, most occurrences had a response time greater than 10 min. Even for events located near ambulance bases (indicated by an ambulance symbol on the map), the response time exceeded 10 min. The posterior exceedance probability for ambulance response time was calculated, as shown in Figure 5. There was a high probability in the central area, indicated by warm colors (0.6–1). In the southeast area, however, the probabilities were lower.

**Figure 6:**
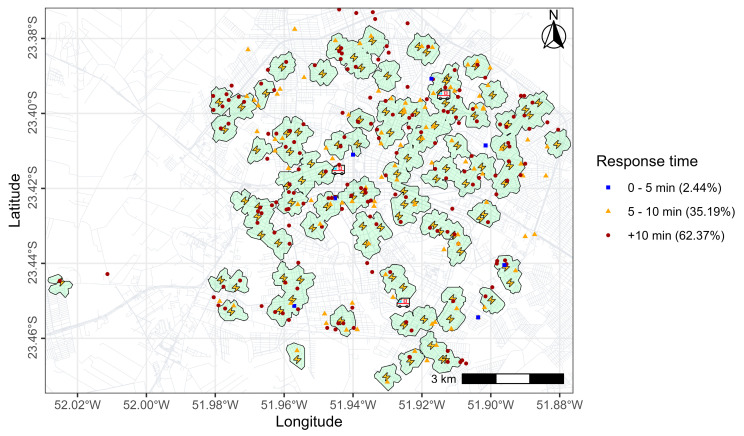
Allocation of AEDs and coverage. The lightning bolt symbol represents the location selected for AED placement.

Figure 6 depicts a map of Maringá, divided into 500 m^2^ cells. Variations in response times seemed to be random throughout the municipality. Nevertheless, there was a higher frequency in regions with mean times between 8 min 20 s and 11 min 40 s, especially from the center to the northeast. Only three cells had a mean response time of less than 5 min.

**Figure 7:**
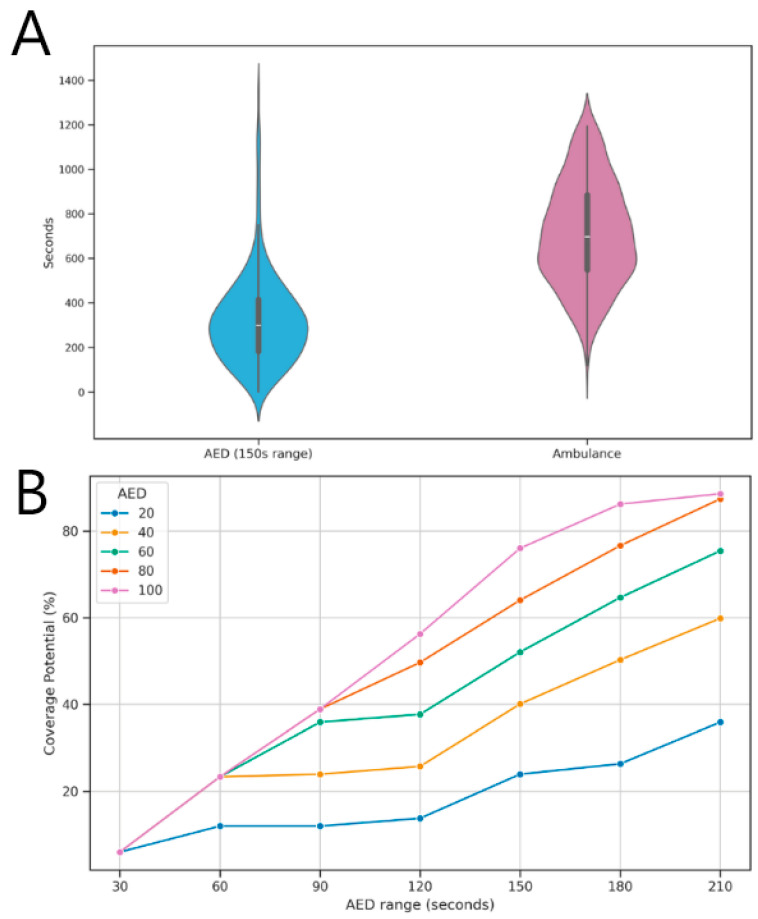
Comparison of coverage times: (**A**) Difference between response times with ambulance and with AED implementation; (**B**) Variation in coverage times with different AED availability.

For a simulated time to defibrillation of 5 min, maximum coverage would be achieved with the allocation of 100 AEDs. Figure 7A illustrates the areas covered by the 100 candidates selected by the model, covering 127 of the 167 demand centroids (76%) within the 5 min response time. On average, the travel time between each OHCA occurrence and the nearest selected candidate was estimated to be 5 min 20 s (320 s), (SD = 201.8 s), with a median of 4 min 59 s (299 s). SAMU response times averaged 11 min 49 s (709 s), (SD = 228.4 s), with a median of 11 min 38 s (698 s) (549–886 s). The Wilcoxon test showed significant differences between medians (z = −13.324 and *p* < 0.01). This finding suggests that, with the installation of AEDs, the response time would be significantly lower than the current time achieved by SAMU.

Figure 7B shows the variation in potential coverage at different time intervals with the increase in AEDs number (increments of 20 units). It was predicted that, with response times of 2.5 min (150 s) and 100 AEDs, it would be possible to cover 76% of the centroids.

## 4. Discussion

To the best of our knowledge, this is the first study in Brazil to demonstrate the impact of SAMU response time for CPR care and simulate the effect of allocating AEDs to urban areas on OHCA outcomes. In the evaluated period, 89 (31.01%) individuals experiencing OHCA had shockable rhythm and were subjected to defibrillation. In 149 (51.92%) occurrences, bystanders initiated CPR, and, in 221 (77%) events, the outcome was death. The median response time was 11 min 38 s (698 s). Some regions were found to be at an increased risk of not being attended by an ambulance within 5 min (300 s). The analysis selected 100 public establishments as candidates to receive AEDs. The median travel time between OHCA occurrence and AED candidate site was 4 min 59 s (299 s), significantly shorter than the ambulance response time.

Studies have demonstrated that CPR and the use of an AED by bystanders can increase the chances of survival by more than three times [36,37]. In this study, only 51.92% of bystanders performed CPR before SAMU arrival, a percentage lower than that found in other studies [23,38]. Bystander use of public access AEDs during OHCA is poor at 2% to 3% [39]. A study performed in Denmark has shown that the probability of bystander defibrillation was low in residential locations [40]. This finding underscores the need for population training, given that about 59% of OHCAs occur in residences [41].

In this study, the SAMU response time was greater than 5 min (300 s) in the majority of cases. Early defibrillation, that is, within 5 min (300 s), can improve survival rates to 50–70%. AEDs availability in easily accessible public areas also contributes to better outcomes [16,42,43,44].

The present study proposed the strategic allocation of 100 AEDs in public areas, which would allow achieving a mean time to defibrillation of 5 min 20 s (320 s). This time is significantly lower than the SAMU response time, by nearly half. The AEDs would be located at a distance that could be reached in 2.5 min (150 s) by bystanders. However, the use of this equipment would require training the population on its proper use and disclosure of their location. Mobile applications could be an effective solution in this regard, providing information on the location of the nearest AED and instructional videos on how to properly operate the equipment [45].

Approximately 4000 lives could be saved annually if public-access AEDs were used in all OHCAs, according to a study conducted in the United States [46]. In the studied municipality, there is only one law mandating the presence of AEDs in places with large concentrations of people [47]. Given that public-access AEDs are a cost-effective public health intervention, laws regulating the implementation of these devices and CPR training for the population are fundamental to improving OHCA outcomes [48].

Increasing the proportion of AEDs used on adult individuals with OHCA before EMS arrival to >20% is one of the American Heart Association Emergency Cardiovascular Care (AHA ECC) Committee’s 2030 Impact Goals [49]. A study has shown that using drones for automated AED delivery in areas with physical barriers is feasible and leads to shorter defibrillation times [50]. In Sweden, AED-equipped drones dispatched in cases of suspected OHCA delivered AEDs before ambulance arrival, providing a clinically relevant median time benefit of more than 3 min (180 s) [51].

Reducing EMS response times can also benefit from technology, such as mobile applications. A group of Brazilian researchers developed an application called CHAMU192 to streamline the call-taking process by accurately identifying the caller’s location. The use of this app by EMS significantly reduced response times [52].

The survival rate of OHCAs increases with early EMS notification and the early application of basic life support (BLS) techniques and defibrillation. In the Netherlands, a text message alert system for trained volunteers was implemented to reduce response times, effectively increasing survival rates and reducing disability in OHCA victims [53].

The use of applications can also contribute to improving records of services currently performed manually in most EMS services in Brazil.

### 4.1. Limitations

This study has some limitations. SAMU response times were recorded manually, and it is not uncommon for operators to record times at the end of the occurrence. This can lead to errors, as operators may not accurately remember the exact time. To circumvent this problem and increase data reliability, it was decided to exclude response times greater than 20 min. Failure in recording incidents can also increase response time. In Brazilian EMS, the majority of first aid reports (RAS) are completed manually on printed forms, making it difficult to create an epidemiological database and preventing the destination hospital from accessing RAS data before the patient’s arrival. Additionally, communication between the EMS team and the destination hospital is conducted by telephone, which can result in the loss of crucial information during this process.

### 4.2. Future Prospects

The candidate establishments to receive AEDs were predominantly located in the central region of Maringá, leaving the most distant areas potentially unassisted. Nevertheless, OHCAs were mainly concentrated in the urban area, where most of the population resides. Therefore, the installation of AEDs in these areas may benefit a large portion of residents. In the future, it will be necessary to reassess the allocation of AEDs and relocate them as needed.

## 5. Conclusions

This study showed that SAMU response times for OHCA in the country tend to frequently exceed the ideal stipulated time of 5 min. The strategic allocation of AEDs is essential for early defibrillation and improving OHCA outcomes. The results provide robust evidence to strengthen the emergency care network throughout Brazil.

## Figures and Tables

**Table 1:** Descriptive analysis of services provided by SAMU between 2019 and 2022 in Maringá, Paraná.

Variables	Descriptive
Age (median in years, IQR)	69 (59, 78)
Response time (median in seconds, IQR)	698 (549, 886)
CPR duration (median in seconds, IQR)	1800 (1200, 2400)
Dispatch time (median in seconds, IQR)	446 (314, 610)
Cardiopulmonary resuscitation performed by bystanders (absolute and relative frequency)	
Yes	149 (51.92%)
No	126 (43.90%)
No information	12 (4.18%)
Gender (absolute and relative frequency)	
Male	168 (58.54%)
Female	119 (41.46%)
Death (absolute and relative frequency)	
Yes	221 (77%)
No	66 (23%)
Shockable rhythm	
Yes	89 (31.01%)
No	198 (68.99%)
SAMU response time (absolute and relative frequency)	
0–5 min	7 (2.44%)
5–10 min	101 (35.19%)
>10 min	179 (62.37%)
Shockable rhythm at ambulance arrival time (absolute and relative frequency)	
0–5 min	2 (2.24%)
5–10 min	37 (41.57%)
>10 min	50 (56.18%)
IQR: Interquartile Range	

## Data Availability

See https://doi.org/10.6084/m9.figshare.c.7373818.v1.
